# F126. PATHWAYS FROM SPEECH ILLUSIONS TO PSYCHOTIC SYMPTOMS IN SUBJECTS AT ULTRA-HIGH RISK FOR PSYCHOSIS: COMBINING AN EXPERIMENTAL PARADIGM OF ABERRANT EXPERIENCES WITH NETWORK ANALYSIS

**DOI:** 10.1093/schbul/sby017.657

**Published:** 2018-04-01

**Authors:** Adela-Maria Isvoranu, Lindylou Boyette, Frederike Schirmbeck, Eva Velthorst, Claudia Simons, Denny Borsboom, Lieuwe De Haan

**Affiliations:** 1 University of Amsterdam; 2 Icahn School of Medicine at Mount Sinai; 3 GGzE / Maastricht University; 4 AMC-Academisch Psychiatrisch Centrum

## Abstract

**Background:**

One of the oldest and most influential theories of psychosis formation states that delusions arise in an attempt to explain unusual experiences, including perceptual aberrations. The White Noise Task by Galdos et al (2011) was developed as an experimental task to assess the tendency to attribute meaning to random perceptual stimuli: speech illusions in white noise. Studies to date have demonstrated that speech illusions as assessed with the White Noise Task are associated with a composite measure of positive symptoms in patients with psychotic disorders (Galdos et al, 2011; Catalan et al, 2014). However, findings in non-clinical samples have been inconsistent: one study found an association with a composite measure of subclinical positive symptoms, including support for a relation with familial psychosis liability (Galdos et al, 2011), whereas other studies did not find any association in non-clinical samples or only partly (Catalan et al, 2014; Rimvall et al, 2016; Pries et al, 2017). The current study aims to further examine whether speech illusions as assessed with the White Noise Task are indicative of psychosis liability and to explore specific symptomatic pathways.

**Methods:**

We conducted symptom-based network analyses in Ultra-High Risk (UHR) subjects participating in the European network of national networks studying gene-environment interactions in schizophrenia project (EU-GEI, 2014; www.eu-gei.eu). Psychotic symptoms were assessed with the Brief Psychiatric Rating Scale (BPRS). Transition to clinical psychosis was assessed with the Comprehensive Assessment of At Risk Mental State (CAARMS). We used a conservative measure of speech illusions, as described in Catalan et al (2014).

**Results:**

The current sample consisted of 339 UHR subjects, of which 9.1% (N=31) experienced speech illusions. Preliminary network analyses in cross-sectional baseline data showed potential pathways from speech illusions to delusional ideation, through hallucinatory experiences. We also found evidence of prospective relations between speech illusions at baseline and transition to clinical psychosis. Pathways ran via baseline psychotic symptoms and affective symptoms, as well as a ‘direct’ pathway.

**Discussion:**

As far as we are aware, this is the first study combining an experimental measure of aberrant experiences with symptom-based network analysis. Although the current reported findings are preliminary and exploratory, they tentatively support a relation between speech illusions as assessed with the White Noise Task and psychosis liability. This relation may be dependent on sample composition, and not generalizable to the general population as a whole. Future studies may benefit from focusing on more detailed trajectories of both susceptibility to speech illusions and course of (sub)clinical psychotic symptom severity in subjects with increased risk for psychosis, with use of more frequent, short assessment periods and inclusion of environmental risk factors for transition to clinical disorder.

